# Impact of intensified prevention measures on rates of hospital-acquired bloodstream infection in medical-surgical intensive care units, Israel, 2011 to 2019

**DOI:** 10.2807/1560-7917.ES.2023.28.25.2200688

**Published:** 2023-06-22

**Authors:** Debby Ben-David, Azza Vaturi, Liat Wulffhart, Elizabeth Temkin, Ester Solter, Yehuda Carmeli, Mitchell J Schwaber, the National HA-BSI Prevention Working Group, Members of the National HA-BSI Prevention Working Group, Hiba Zayyad, Kozita Libai, Alona Paz, Mirit Hershman-Sarafov, Bibiana Chazan, Iris Grinberg Avraham, Tal Brosh Nisimov, Nir Maaravi, Maya Kats, Shmuel Benenson, Carmela Schwartz, Ilana Gross, Sarah Israel, Yonatan Oster, Kami Harpaz, Ronza Najjar-Debbiny, Gabriel Weber, Pnina Shitrit, Alia Yassin, Ortal Hilel, Bina Rubinovitch, Pierre Singer, Hefziba Madar, Khetam Hussein, Halima Dabaja-Younis, Tamar Alon, Yonit Wiener-Well, Liora Bier, Lili Goldshtein, Dan Klafter, , Regev Cohen, Danielle Atiya,, Asaf Biber, Elena Machtin, Iris Zohar, Yael Cohen

**Affiliations:** 1 National Center for Infection Control, Ministry of Health, Jerusalem, Israel; 2 Sackler Faculty of Medicine, Tel Aviv University, Tel Aviv, Israel; 3 The members of the National HA-BSI Prevention Working Group are listed at the end of the article

**Keywords:** CLABSI, national intervention, surveillance, hospital acquired bloodstream infections

## Abstract

**Background:**

Central line-associated bloodstream infection (CLABSI) is among the most common preventable infectious complications in patients in intensive care units (ICU). In 2011, the Israel National Center for Infection Control initiated a nationwide CLABSI prevention programme.

**Aim:**

To evaluate the impact of different components of the programme on CLABSI and non-CLABSI rates in medical-surgical ICUs.

**Methods:**

We included data collected from all 29 medical-surgical ICUs in Israel from November 2011 to December 2019. The study period was divided into three phases: I (baseline, initial CLABSI prevention guidelines introduced, initial feedback on rates provided), II (initial guidelines widely implemented, surveillance undertaken, feedback continued) and III (after implementation of additional prevention measures). Interrupted time series analysis was used to compare CLABSI and non-CLABSI rates during the three phases.

**Results:**

The pooled mean (SD) incidence of CLABSI per 1,000 central line-days dropped from 7.4 (0.38) in phase I to 2.1 (0.13) in phase III (p < 0.001). The incidence rate ratio (IRR) was 0.63 (95% CI: 0.51–0.79) between phases I and II, and 0.78 (95% CI: 0.59–1.02) between phases II and III. The pooled mean (SD) incidence of non-CLABSI per 1,000 patient-days declined from 5.3 (0.24) in phase I to 3.4 (0.13) in phase III (p < 0.001).

**Conclusion:**

National CLABSI prevention guidelines, surveillance and feedback resulted in significant reductions in CLABSI and non-CLABSI rates. In the wake of further interventions, significant reduction was achieved in ICUs reporting improvement in the uptake of additional prevention measures.

**Key public health message**

**What did you want to address in this study?**

During the past 15 years, national and regional interventions have reportedly led to a marked reduction in central line-associated bloodstream infections (CLABSI), yet sparse data exist on the impact on total hospital-acquired BSI (HA-BSI). We assessed the impact of different components of a national programme on the incidence of CLABSI and total HA-BSI in intensive care units in Israel.

**What have we learnt from this study?**

Over 8 years, a national intervention conducted in Israeli ICUs prevented ca 2,200 episodes of HA-BSI, including 1,300 CLABSI events. The reduction in CLABSI rates was observed in two stages. An initial reduction occurred shortly after implementation of prevention bundles, infection surveillance and feedback. Following introduction of additional measures, a further reduction was observed.

**What are the implications of your findings for public health?**

The rate of total HA-BSI may be a more objective measure of hospital safety than CLABSI rates alone. Furthermore, CLABSI events account for only 25–35% of all HA-BSI. National surveillance programmes should thus monitor additional causes of preventable HA-BSI. Assessment of prevention measures may uncover the reasons for variation in infection rates between facilities and reveal gaps in knowledge and resources.

## Introduction

Hospital-acquired bloodstream infections (HA-BSI) are among the most common infections acquired by patients in intensive care units (ICUs), affecting ca 5–7% of patients admitted to ICUs [1,2]. Risk factors for HA-BSI include severeness of illness at admission, prolonged length of stay, immunosuppression and the presence of central venous catheters [2]. Hospital-acquired bloodstream infections are associated with high mortality rates, increased length of ICU stay and healthcare-related costs [3]. Findings from surveillance conducted by the World Health Organization indicate high rates of antibiotic resistance among pathogens causing bloodstream infections (BSI) [4], which may contribute to adverse patient outcomes. Bloodstream infections caused by drug-resistant pathogens are associated with higher rates of 1-year mortality compared with BSI caused by susceptible pathogens [5].

Hospital-acquired bloodstream infections may be primary or secondary to the dissemination of pathogens from another anatomic site. Primary BSI are laboratory-confirmed BSI that are not related to infection at another body site [6]. In the presence of a central venous line, primary BSI are defined as central line-associated bloodstream infections (CLABSI). In a large European survey, 39.5% of bloodstream infections were central venous catheter-related [7]. The most common sources of secondary HA-BSI are respiratory and urinary tracts [8].

During the past 15 years, numerous national and regional programmes have focused on prevention of CLABSI. Prevention bundles, such as those first proposed by Pronovost et al. in 2006 [9], have become the standard of care for CLABSI prevention. Several multifaceted interventions subsequently conducted worldwide have resulted in a marked reduction in CLABSI rates [10].

The Israel National Center for Infection Control (NCIC) initiated a nationwide CLABSI prevention programme in 2011. The primary aim of this study is to evaluate the impact of different components of the programme on the incidence of CLABSI. Secondary aims are to determine the impact of the intervention programme on rates of non-CLABSI and total ICU-acquired BSI and to evaluate factors associated with improvement.

## Methods

### Design and setting

This was a prospective, non-randomised nation-wide intervention. The setting was general ICUs in all 29 of Israel’s acute care hospitals, which were divided into three categories: tertiary care, medium-sized non-tertiary care (≥ 400 beds) and small non-tertiary care (< 400 beds).

**Figure 1:**
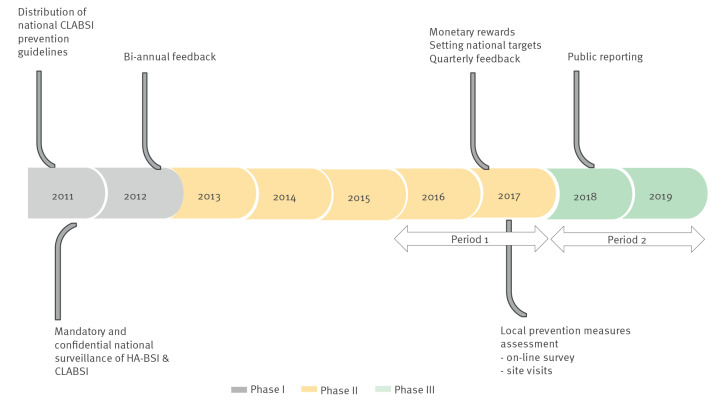
Timeline of the national interventions implemented in general intensive care units, Israel 2011–2019

HA-BSI: hospital-acquired bloodstream infections; CLABSI: central line-associated bloodstream infections.

The study consisted of three phases: (i) phase I, baseline, upon introduction of initial prevention measures (November 2011–December 2012); (ii) phase II, after initial measures were widely implemented, surveillance undertaken and routine feedback provided to hospitals (January 2013–December 2017); (iii) phase III, after hospitals had adopted additional prevention measures (January 2018–December 2019) (Figure 1).

### Intervention I

Central line-associated bloodstream infection prevention guidelines were distributed to acute care hospitals in May 2011. In November 2011, mandatory, confidential national surveillance of HA-BSI and CLABSI was initiated. In addition, workshops for infection control and ICU staff were conducted by the NCIC throughout the intervention period. Biannual feedback on infection rates were send to hospital management and local infection control teams starting in August 2012.

### Intervention II

During 2017, in response to a plateau in CLABSI rates, the following additional steps were implemented: (i) a target of achieving below the 25th percentile of the 2016 incidence rate was set; (ii) frequency of feedback was increased to quarterly; (iii) site visits were conducted by NCIC staff, during which local strategies were evaluated with infection control and ICU teams. Additional interventional elements included the following: (i) an infection control incentive programme launched by the Ministry of Health in 2016 that included monetary rewards to hospitals based, in part, on meeting national standards for CLABSI rates; (ii) the release of hospital-level data on CLABSI rates to the public from 2018; and (iii) simulations for ICU staff encompassing central line insertion and maintenance.

In June 2017, an online survey assessing CLABSI prevention practices in general ICUs was sent to hospital infection control teams. Questions focused on prevention measures within two domains: (i) technology usage (use of ultrasound, alcohol caps, chlorhexidine and insertion carts); and (ii) implementation methods (education, audits and ward champions) [11]. A follow-up survey was sent in June 2018. Each hospital received feedback with the summary of local implemented measures compared with other facilities and correlated with CLABSI incidence.

### Surveillance

National BSI surveillance was launched in November 2011 using the United States (US) Centers for Disease Control and Prevention (CDC) surveillance definitions and methodologies, which were updated annually [6]. Trained infection preventionists in each facility collected data on ICU patients. From November 2011 to December 2015, hospitals reported aggregate data monthly. Intensive care unit-acquired BSI cases were classified as CLABSI or non-CLABSI, where non-CLABSI referred to cases not associated with a central line. As of May 2016, patient-level data replaced aggregate reports.

The data included all positive blood cultures, admission and discharge dates, symptoms and signs, dates of diagnostic procedures and presence of central venous lines. All positive blood cultures were classified into three categories: (i) contamination, (ii) present on admission and (iii) HA-BSI. Hospital-acquired bloodstream infections were further classified as CLABSI, primary non-CLABSI or secondary BSI. All reports were reviewed and validated by an infection preventionist at the NCIC, to ensure that the cases were classified in accordance with CDC definitions. Whenever a discrepancy was found between an institution’s surveillance assessment and a CDC definition, the case was clarified with the hospital infection control team.

Total HA-BSI and CLABSI incidence rates were calculated as the number of infections per 1,000 patient-days and 1,000 line-days, respectively. Device utilisation was defined as the ratio of total central line-days to total patient-days.

### Statistical analysis

Using the aggregate data collected since 2011, trends in CLABSI and non-CLABSI rates were compared between the three phases. We conducted an interrupted time series analysis to estimate the effect of the two rounds of interventions on CLABSI incidence rates. The number of cases prevented was estimated by comparing the expected number of CLABSI events based on the 2012 rate to the observed number in years 2013–2019.

Using the expanded patient-level data collected since mid-2016, which allowed us to verify hospitals’ classification of BSI as CLABSI or non-CLABSI and to assess the sources of non-CLABSI events, we compared the incidence rates between two periods: (i) period 1, before the adoption of additional prevention measures (May 2016–December 2017); and (ii) period 2, after the adoption of additional prevention measures (January 2018–December 2019).

We divided pathogens into the following four categories: (i) common Enterobacterales (*Klebsiella pneumoniae*, *Escherichia coli*, *Enterobacter* spp.); (ii) non-fermenting Gram-negative bacteria (*Pseudomonas aeruginosa* and *Acinetobacter* spp.); (iii) Gram-positive bacteria (*Enterococcus* spp., *Staphylococcus aureus*, coagulase-negative staphylococci); and (iv) *Candida* spp. All pathogens not included in the prior four categories were grouped as ‘other’. The pooled mean pathogen-specific incidence per 10,000 patient-days was calculated for primary (CLABSI and non-CLABSI) and secondary BSI events. Incidence rate difference (IRD) between periods was calculated.

For each round of surveys, hospitals were assigned a prevention score based on the number of CLABSI prevention measures that they had implemented. The association between the prevention score and CLABSI rates during the first and second surveys was assessed using Spearman’s correlation. Student’s t-tests were used to compare uptake of prevention measures at baseline and follow-up. Hospitals were categorised into two groups based on high (> median score on the first survey) or low (≤ median score on the first survey) uptake of prevention measures. Incidence rate ratio (IRR) was used to compare CLABSI rates between periods 1 and 2 separately for hospitals with high and low uptake of prevention measures at baseline. Significance was set at p < 0.05. Data were analysed using Python version 3.7.4 (Python Software Foundation, Wilmington, US) and Rstudio version 3.6.3 (Posit Software, Boston, US).

## Results

### Central line-associated bloodstream infection and non-central line-associated bloodstream infection rates

**Figure 2:**
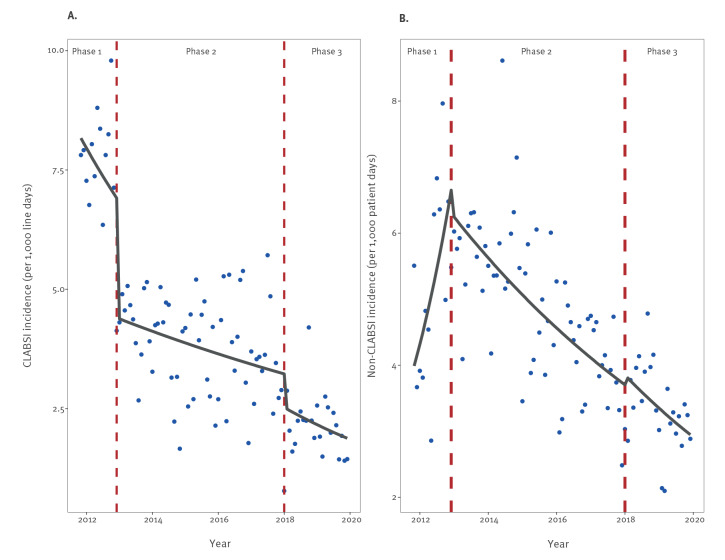
Trends in (A) pooled mean central line-associated bloodstream infection and (B) pooled mean non-central line-associated bloodstream infection rates in intensive care units, Israel, 2012–2019

The Table summarises CLABSI and non-CLABSI incidence rates and central venous catheter use by year. As shown in Figure 2, the mean (standard deviation (SD)) pooled monthly incidence of CLABSI per 1,000 central line-days dropped from 7.4 (0.38) in phase I, to 3.8 (0.11) in phase II, to 2.1 (0.13) in phase III (p < 0.001 for phase I vs phase III). Incidence rate ratio was 0.63 (95% CI: 0.51–0.79) between phases I and II, and 0.78 (95% CI: 0.59–1.02) between phases II and III.

**Table:** Central line-associated bloodstream infections and non-central line-associated bloodstream infections in intensive care units, Israel, 2011–2019

Variable	Phase I				Phase II										Phase III			
	2011		2012		2013		2014		2015		2016		2017		2018		2019	
Reporting ICUs (n)	17		27		26		27		27		28		29		29		29	
Patient-days	8,992		79,394		90,552		92,510		96,611		81,811		99,483		104,550		109,014	
Central line days	3,938		47,105		57,730		58,577		58,446		50,825		59,721		60,529		63,115	
Device utilisation ratio	0.44		0.59		0.64		0.63		0.60		0.62		0.60		0.58		0.58	
CLABSI (n)	31		349		251		218		217		197		210		134		127	
Non-CLABSI (n)	40		428		516		541		458		346		393		389		333	
Total ICU-acquired BSI (n)	71		777		767		759		675		543		603		523		460	
Pooled mean	n	95% CI	n	95% CI	n	95% CI	n	95% CI	n	95% CI	n	95% CI	n	95% CI	n	95% CI	n	95% CI
CLABSI^a^	7.9	5.1 to 10.6	7.4	6.6 to 8.2	4.3	3.8 to 4.9	3.7	3.2 to 4.2	3.7	3.2 to −4.2	3.9	3.3 to 4.4	3.5	3.6 to 4.0	2.2	1.8 to 2.6	2.0	1.7 to 2.4
Non-CLABSI^b^	4.4	3.1 to 5.8	5.4	4.9 to 5.9	5.7	5.2 to 6.2	5.8	5.4 to 6.3	4.7	4.3 to 5.2	4.2	3.8 to 4.7	4.0	3.6 to 4.3	3.7	3.4 to −4.1	3.1	2.7 to −3.4
Total ICU-acquired BSI^b^	7.6	5.8 to 9.4	9.3	8.7 to 10.0	8.4	7.8 to 9.0	8.2	7.6 to −8.8	7.0	6.5 to 7.5	6.6	6.0 to 7.1	6.1	5.6 to 6.5	5.0	4.6 to 5.4	4.2	3.8 to 4.6

BSI: bloodstream infection; CI: confidence interval; CLABSI: central line associated bloodstream infection; ICU: intensive care unit.

^a^ Per 1,000 central line-days.

^b^ Per 1,000 patient days.

CLABSI: central line associated bloodstream infection.

The mean (SD) pooled monthly incidence of non-CLABSI per 1,000 patient-days declined from 5.3 (0.24) in phase I, to 4.9 (0.1) in phase II, to 3.4 (0.13) in phase III (p < 0.001). Beginning in 2013, non-CLABSI incidence decreased steadily (i.e. unrelated to the intervention phases) by 8.8% per year (95% CI: 6.1–8.8, p < 0.0001).

The mean (SD) pooled monthly incidence of total ICU-acquired BSI per 1,000 patient-days was 9.6 (0.33), 7.3 (0.13) and 4.6 (0.15) in phases I, II and III, respectively (p < 0.001). The decrease in HA-BSI rates since November 2011 translates into ca 1,300 CLABSI events and 900 non-CLABSI events prevented between 2013 and 2019.

### Infection source, pathogens, and comparison by hospital size

**Figure 3:**
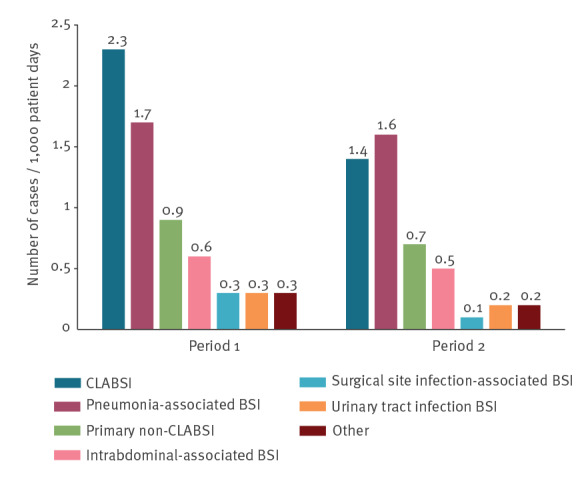
Source of bloodstream infection in intensive care units, Israel, period 1 (May 2016−December 2017) vs period 2 (January 2018−December 2019)

BSI: bloodstream infection; CLABSI: central line-associated bloodstream infection.

From May 2016 to December 2017 (period 1), a total of 1,146 HA-BSIs were recorded. Central line-associated bloodstream infections were the most common cause, accounting for 35.5% (407/1,146) of HA-BSI events. The most common secondary source was the respiratory tract (22.8%, 261/1,146) followed by the abdomen (8.0%, 92/1,146) (Figure 3) The Supplementary Table S1 presents a detailed description of the attributed sources of hospital-acquired bloodstream infections categorized by different types of hospitals.

Central line-associated bloodstream infection rates were significantly lower in tertiary hospitals (3.0, 95% CI: 2.5–3.6) compared with medium-sized (4.2, 95% CI: 3.5–4.8, p = 0.008) and small hospitals (5.3, 95% CI: 4.1–6.1, p < 0.001). In contrast, higher rates of non-CLABSI events were observed in tertiary hospitals, including BSI secondary to pneumonia, intra-abdominal infections and surgical site infections (Supplementary Table S1).

Pooled means of CLABSI and non-CLABSI rates declined significantly between period 1 and period 2: CLABSI 3.7 (95% CI: 3.4–4.1) to 2.1 (95% CI: 1.9–2.4) per 1,000 central line-days p < 0.001; non-CLABSI 4.1 (95% CI: 3.8–4.4) to 3.4 (95% CI: 3.2–3.7) per 1,000 patient-days, p < 0.001. Hospital-acquired bloodstream infections secondary to urinary tract and surgical site infection can be seen in Supplementary Table S1. During the second period, pneumonia replaced CLABSI as the most common source of HA-BSI (34.4%, (338/983). The decrease in CLABSI rates was larger in medium-sized and small hospitals (IRD: −2.0, 95% CI: −1.3 to −2.8, and −2.7, 95% CI: −1.3 to −4.0, respectively) than in tertiary hospitals (IRD: −1.03, 95% CI: −0.39 to −1.67) (Supplementary Table S1).

**Figure 4:**
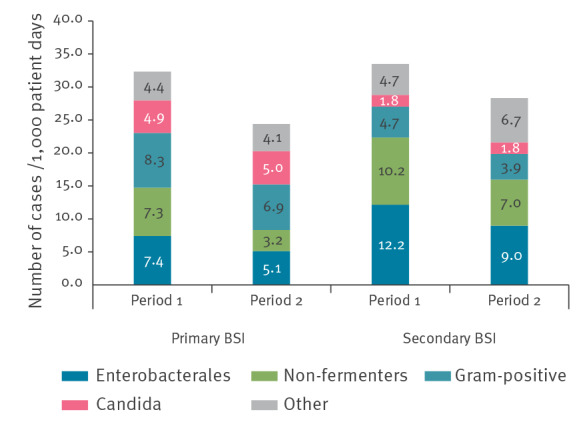
Proportions of pathogens isolated in primary and secondary bloodstream infections in intensive care units comparing period 1 (May 2016–December 2017) vs period 2 (January 2018–December 2019)

Figure 4 depicts proportions of pathogens isolated in the two intervention periods. Gram-negative bacteria were most common. There was a significant decrease in primary and secondary BSI caused by Enterobacterales (IRD: −2.27, 95% CI: −0.67 to −3.8, IRD: −3.7, 95% CI: −1.9 to −5.26, respectively) and non-fermenting Gram-negative bacteria (IRD: −4.15, 95% CI: −2.71 to −5.59, IRR: −3.2, 95% CI: −1.33 to −5.07, respectively). No change was observed in HA-BSI caused by *Candida* spp. (IRD: 0.11, 95% CI: −1.34 to 1.56, IRR: −0.01, 95% CI: −0.88 to 0.85) and Gram-positive bacteria (IRR: −1.39, 95% CI: −3.17 to 0.38, IRR: −0.80, 95% CI: −2.13 to 0.54, respectively).

BSI: bloodstream infection.

The figures in the bars represent number of cases per 1,000 patient days.

### Comparison of uptake of prevention measures and association with central line-associated bloodstream infection rates

**Figure 5:**
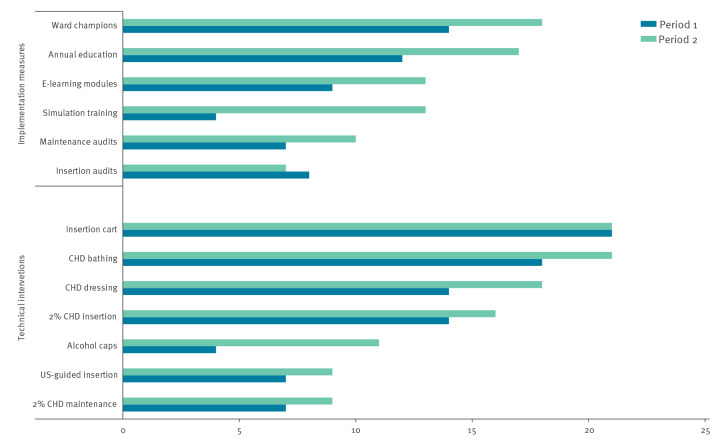
Uptake of infection control measures in intensive care units, Israel, period 1 (May 2016–December 2017) vs period 2 (January 2018–December 2019)

CHD: chlorhexidine; US: ultrasound.

Twenty-one of 29 acute care hospitals completed both surveys. Figure 5 illustrates the uptake of different prevention measures in the two survey rounds. The most widely used prevention measures were dedicated supply carts for central line insertion, routine chlorhexidine (CHD) bathing and CHD-impregnated dressings at the insertion site. The greatest increase between the surveys was found for performing simulation training and use of alcohol caps. Fewer than half of the facilities reported routine use of ultrasound during central line insertion or conducting audits on insertion and maintenance practices.

The mean (SD) number of prevention measures increased from 7.3 (2.9) in the baseline survey to 9.9 (2.0) in the follow-up survey (p = 0.002). In the first period, the pooled CLABSI incidence rate was higher in the 14 ICUs with low prevention scores (4.4 per 1,000 line-days) than in the seven ICUs with high prevention scores (2.9 per 1,000 central line-days) (IRR: 1.5, 95% CI: 1.2–2.0, p < 0.001). In the group of hospitals with low prevention scores at baseline, the mean number of prevention measures used increased from 6.0 at baseline to 9.9 at follow-up (p < 0.001), and the pooled CLABSI incidence rate in this group decreased from 4.4 to 1.9 per 1,000 line-days (IRR: 0.44, 95% CI: 0.32–0.59, p < 0.001). In hospitals with high prevention scores at baseline, neither the mean score (10 vs 9.8) nor the pooled CLABSI rate (2.9 vs 2.3) changed significantly between the two periods.

## Discussion

During a period of 8 years, a national intervention conducted in medical-surgical ICUs in Israel achieved a significant and sustained reduction in both CLABSI and non-CLABSI rates. The reduction in CLABSI rates was observed in two stages. The first reduction occurred shortly after widespread implementation of a national evidence-based prevention bundle, infection surveillance and feedback. Subsequently, during a period of 5 years, no significant change was observed in CLABSI rates. In 2018, following a multifaceted intervention, a further reduction was observed. The reduction in CLABSI rates was likely achieved by the multimodal intervention approach taken, which included feedback to hospitals on adoption of prevention measures in comparison with other hospitals and the inclusion of CLABSI rates in a nationwide incentive programme and public reporting. Of note, a significant reduction was achieved only in ICUs reporting improvement in the uptake of additional prevention measures. Additionally, although the national programme was initially focused on CLABSI-targeted interventions, the continuous measurement and feedback on all cases of HA-BSI led to a progressive and sustained reduction in non-CLABSI rates.

The seminal 1988 *Study on the Efficacy of Nosocomial Infection Control (SENIC)* demonstrated that surveillance is a fundamental tool for preventing hospital-acquired infections [12]. Since the SENIC study, implementation of national surveillance systems has led to a marked reduction in CLABSI rates [13-15]. Nevertheless, CLABSI rates may remain high despite implementation of surveillance programmes [16]. Variability in the use of prevention measures, failure to assess compliance and insufficient involvement of hospital management all likely contribute to suboptimal outcomes [17]. Central line-associated bloodstream infection rates in resource-limited countries are 3–5 times higher than those in high-income settings [14,18]. Lower compliance with insertion and maintenance prevention measures was reported in middle-income countries compared with high-income countries [19].

National assessment of prevention measures may uncover the reasons for variation in infection rates between facilities and reveal gaps in knowledge or resources. In our previous study, we found an association between a high prevention score and low CLABSI rates [11]. Tertiary hospitals, where CLABSI rates were lowest, reported greater use of prevention measures compared with other hospital categories. High infection rates in smaller hospitals may reflect the lack of infection control personnel and resources [14].

The follow-up assessment revealed an increase in uptake of prevention measures and an accompanying decrease in CLABSI rates. This increase was observed in facilities with low prevention scores in the first assessment. Most hospitals have implemented the use of certain technology measures (e.g. insertion cart or CHD dressing). However, other prevention tools, such as simulation training and line-care audits, were adopted to a lesser extent. Hands-on training through simulation was found to be more effective than traditional education for teaching sterile technique and ultrasound skills [20]. In addition, high compliance with insertion and maintenance bundles was found to be associated with low CLABSI rates [21]. Future regional and national interventions should incorporate simulation training and routine measurement of compliance with the bundle elements.

Central line-associated bloodstream infection rates are increasingly published in US healthcare facilities and the data are available to consumers, healthcare providers and hospital administrators and enable comparisons of hospital performance [22]. However, poor inter-rater agreement in classifying infection events makes it difficult to reliably compare rates at different facilities [23]. Furthermore, underestimation of true CLABSI incidence has been found in publicly reported data, diminishing the validity of surveillance measures [24]. Thus, national surveillance programmes should incorporate regular external validation methods to ensure the accuracy of the reported data. One of the strengths of our surveillance programme is that all positive blood cultures are reported and validated. We therefore believe that the decrease in HA-BSI events we found is real and not an artefact of misclassification or under-reporting.

Total hospital-acquired BSIs may be a more objective measure of hospital safety than CLABSI, as there is no classification involved. In 2011, to avoid misclassification of CLABSI events, Israel mandated reporting of all HA-BSI. In all the study years, CLABSI accounted for only 25–35% of all HA-BSI, indicating that national surveillance programmes should include additional sources of preventable HA-BSI. Notably, during the study period, in addition to a decrease in CLABSI rates, we observed a persistent decrease in non-CLABSI events. The significant decrease in non-CLABSI rates was not related to the timing of targeted CLABSI control measures but may be attributable to other concurrent interventions. For example, throughout the study period there was intense national activity directed towards reducing hospital-acquired infections, including catheter-associated urinary tract infections and prevention of carbapenem-resistant Enterobacterales infection and colonisation [25]. Classification of non-CLABSI events is essential to identify intervention targets. Since 2016, the individualised reporting system enabled us to review all positive BSI classifications including contaminants, secondary and primary BSI events. Most HA-BSI events were associated with medical devices, including ventilators and central venous catheters. Intensified interventions to reduce ventilation days and urinary catheter use can be expected to have further impact on HA-BSI.

In the current study, Gram-negative bacteria were found to comprise a sizable proportion of primary BSI, accounting for ca 39.0% (413/1,059) of cases. Gram-positive bacteria were involved in only 26.4% (280/1,059) of events. During recent years, Gram-negative bacilli and *Candida* spp. have become leading causes of primary BSI [26,27]. In our study, most pathogen-specific CLABSI rates decreased over time, with the exception of *Candida* spp. Central line insertion and maintenance bundles may be less effective at preventing bloodstream infections caused by *Candida*. Current CDC definitions, however, may also influence pathogen distribution. *Candida* spp. are not eligible to be considered causative of urinary tract infection or pneumonia [6]. Consequently, most candidaemia events will be defined as primary BSI. Understanding trends in pathogen epidemiology may help in the development of prevention measures tailored to specific pathogens.

The strengths of this study are that it is at country level, and that, since May 2016, all the reported data have been validated. The study has a number of limitations. It was an observational study, lacking a control group. Therefore, it is difficult to determine whether there are factors other than the intervention that contributed to the reduction in CLABSI and HA-BSI rates. Furthermore, implementation of prevention measures was assessed using questionnaires rather than direct observations in each facility. An additional limitation is that the frequency of blood culture collection may have an impact on the incidence of HA-BSI [28]. We did not collect data on the monthly rate of blood cultures obtained in each unit. The study lacks demographic characteristics of the patients. However, individual risk factors have been demonstrated to be less important than infection control practices in determining CLABSI rates. For example, following the implementation of enhanced care bundles, a sustained decrease in CLABSI rates was achieved despite a continuous increase in underlying disease severity over time [29]. Finally, while division of the study period into discreet phases based on timing of policy initiatives is necessary for purposes of analysis, actual implementation of policy changes across multiple institutions occurs gradually and at varying paces based on the respective hospital. Therefore, the true, ultimate effect of our intervention on BSI events prevented may be greater than that reflected in the statistical analysis.

## Conclusion

We found significant reductions in CLABSI and non-CLABSI rates over a period of 8 years in the context of a national intervention, associated with implementation of specific prevention measures at different points in time. A large proportion of the non-CLABSI events were associated with invasive devices and may be preventable.

## Members of the National HA-BSI Prevention Working Group

**The Baruch Padeh Medical Center, Poriya**  

Hiba Zayyad, Kozita Libai

**Bnai Zion Medical Center, Haifa**  

Alona Paz, Mirit Hershman-Sarafov  

**Ha'Emek Medical Center, Afula** 

Bibiana Chazan, Iris Grinberg Avraham 

**Samson Assuta Ashdod University Hospital, Ashdod **

Tal Brosh Nissimov, Nir Maaravi, Maya Kats

**Hadassah Hebrew University Medical Center, Jerusalem**  

Shmuel Benenson, Carmela Schwartz, Ilana Gross

Sarah Israel, Yonatan Oster, Kami Harpaz

**Lady Davis Carmel Medical Center, Haifa** 

Ronza Najjar-Debbiny, Gabriel Weber 

**Meir Medical Center, Kfar Saba** 

Pnina Shitrit, Alia Yassin, Ortal Hilel

**Rabin Medical Center, Beilinson Hospital, Petah Tikva** 

Bina Rubinovitch, Pierre Singer, Hefziba Madar

**Rambam Health Care Campus, Haifa** 

Khetam Hussein, Halima Dabaja-Younis, Tamar Alon

**Shaare Zedek Medical Center, Jerusalem** 

Yonit Wiener-Well, Liora Bier

**Shamir Medical Center, Beer Yaakov** 

Lili Goldshtein, Dan Klafter

**Sanz Medical Center, Laniado Hospital, Netanya** 

Regev Cohen, Danielle Atiya

**Sheba Medical Center, Ramat Gan** 

Asaf Biber, Elena Machtin

**Wolfson Medical Center, Holon**  

Iris Zohar, Yael Cohen

## Supplementary Data

109000 Supplement
