# Supplementary material for: Impact of intensified prevention measures on rates of hospital-acquired bloodstream infection in medical-surgical intensive care units, Israel, 2011 to 2019
Source: Euro Surveill. 2023 Jun 22;28(25):2200688. doi: 10.2807/1560-7917.ES.2023.28.25.2200688 (PMC10288825; doi:10.2807/1560-7917.ES.2023.28.25.2200688)
Supplement: Supplement [file 22-00688_BEN-DAVID_supplement.pdf]

Title page – Supplementary material

This supplementary material is hosted by *Eurosurveillance* as supporting information alongside the article, 'Impact of intensified prevention measures on rates of hospital-acquired bloodstream infection in medical-surgical intensive care units, Israel, 2011 to 2019' on behalf of the authors, who remain responsible for the accuracy and appropriateness of the content. The same standards for ethics, copyright, attributions, and permissions as for the article apply. Supplements are not edited by *Eurosurveillance* and the journal is not responsible for the maintenance of any links or email addresses provided therein.

Supplementary Table S1:

Attributed sources of hospital-acquired bloodstream infections by hospital category: 2016-2019

		Total BSI # (95% CI)	CLABSI^ (95% CI)	Non-CLABSI# (95% CI)	Primary non-CLABSI #(95% CI)	Pneumonia-associated BSI# (95% CI)	Intrabdominal-associated BSI #(95% CI)	Surgical site infection-associated BSI# (95% CI)	Urinary tract infection-associated BSI # (95% CI)	Other# (95% CI)
All hospitals	Period 1	6.4 (6.0,6.8)	3.7 (3.4, 4.1)	4.1 (3.8, 4.4)	0.9 (0.7, 1.0)	1.7 (1.5, 1.9)	0.6 (0.5, 0.7)	0.3 (0.2,.0,3)	0.3 (0.2, 0.4)	0.3 (0.2, 0.4)
	Period 2	4.7 (4.4,5.0)	2.1 (1.9, 2.4)	3.4 (3.2, 3.7)	0.7 (0.6, 0.8)	1.6 (1.5, 1.8)	0.5 (0.4, 0.6)	0.1 (0.1, 0.2)	0.2 (0.1, 0.2)	0.2 (0.1, 0.2)
	P	<0.001	<0.001	<0.001	0.07	0.76	0.54	0.002	0.01	0.09
Tertiary hospitals	Period 1	6.8 (6.2, 7.4)	3.0 (2.5, 3.6)	4.9 (4.3, 5.4)	0.9 (0.7, 1.2)	1.9 (1.6, 2.2)	0.7 (0.5, 0.9)	0.4 (0.3, 0.6)	0.3(0.1, 0.4)	0.4 (0.3,0.6)
	Period 2	6.0 (5.5, 6.5)	2.0 (1.6, 2.4)	4.7 (4.2, 5.2)	0.8 (0.6, 0.9)	2.3 (1.9, 2.6)	0.8 (0.6, 1.0)	0.2 (0.1, 0.3)	0.2 (0.1, 0.3)	0.3 (0.2,0.5)
	P	0.03	0.002	0.5	0.23	0.11	0.7	0.006	0.66	0.31
Medium-sized hospitals	Period 1	6.3 (5.7,7.0)	4.2 (3.5, 4.8)	3.8 (3.4,4.3)	0.85 (0.6,1.1)	1.6 (1.3,2.0)	0.5 (0.3,0.7)	0.2 (0.1,0.3)	0.4 (0.2,0.5)	0.2 (0.07,0.30)
	Period 2	4.0 (3.6, 4.4)	2.1 (1.7,2.6)	2.8 (2.4,3.1)	0.60 (0.4,0.8)	1.4 (1.2,1.7)	0.4 (0.2,0.5)	0.10 (0.03,0.2)	0.2(0.07,0.3)	0.1 (0.03,0.2)
	P	<0.001	<0.001	0.004	0.16	0.54	0.25	0.30	0.02	0.21
Small hospitals	Period 1	5.8 (4.8, 6.7)	5.3 (4.1, 6.1)	2.9 (2.2, 3.6)	0.8 (0.4, 1.1)	1.1 (0.7, 1.6)	0.4 (0.2, 0.7)	0.1 (0.02, 2.3)	0.2 (0.02,0.38)	0.04 (0.03,0.12)
	Period 2	3.6 (3.0, 4.2)	2.6 (1.9, 3.3)	2.3 (1.8, 2.7)	0.8 (0.5, 1.0)	0.9 (0.6, 1.1)	0.4 (0.2, 0.6)	0.1 (0.002,0.26)	0.1 (0.02, 0.19)	0.07 (0.01, 0.16)
	P	0.002	<0.001	0.42	0.69	0.55	0.81	0.91	0.36	0.52

Per 1000 patient-days
^Per 1000 central line catheter days
CLABSI, central line associated bloodstream infection; CI, confidence interval
